# A rare case of primary clear cell sarcoma of the pubic bone resembling small round cell tumor: an unusual morphological variant

**DOI:** 10.1186/1471-2407-12-538

**Published:** 2012-11-21

**Authors:** Shoko Nakayama, Taiji Yokote, Kazuki Iwaki, Toshikazu Akioka, Takuji Miyoshi, Yuji Hirata, Ayami Takayama, Uta Nishiwaki, Yuki Masuda, Motomu Tsuji, Toshiaki Hanafusa

**Affiliations:** 1 Department of Internal Medicine (I), Osaka Medical College, 2-7 Daigakumachi, Takatsuki City, Osaka, 569-0801, Japan; 2 Division of Surgical Pathology, Osaka Medical College, 2-7 Daigakumachi, Takatsuki City, Osaka, 569-0801, Japan

**Keywords:** Clear cell sarcoma, Small round cell tumor, Pubic bone, Immunohistochemistry

## Abstract

**Background:**

Clear cell sarcoma (CCS) and malignant melanoma share overlapping immunohistochemistry with regard to the melanocytic markers HMB45, S100, and Melan-A. However, the translocation t(12; 22)(q13; q12) is specific to CCS. Therefore, although these neoplasms are closely related, they are now considered to be distinct entities. However, the translocation is apparently detectable only in 50%–70% of CCS cases. Therefore, the absence of a detectable *EWS/AFT1* rearrangement may occasionally lead to erroneous exclusion of a translocation-negative CCS. Therefore, histological assessment is essential for the correct diagnosis of CCS. Primary CCS of the bone is exceedingly rare. Only a few cases of primary CCS arising in the ulna, metatarsals, ribs, radius, sacrum, and humerus have been reported, and primary CCS arising in the pubic bone has not been reported till date.

**Case presentation:**

We present the case of an 81-year-old man with primary CCS of the pubic bone. Histological examination of the pubic bone revealed monomorphic small-sized cells arranged predominantly as a diffuse sheet with round, hyperchromatic nuclei and inconspicuous nucleoli. The cells had scant cytoplasm, and the biopsy findings indicated small round cell tumor (SRCT). Immunohistochemical staining revealed the tumor cells to be positive for HMB45, S100, and Melan-A but negative for cytokeratin (AE1/AE3) and epithelial membrane antigen. To the best of our knowledge, this is the first case report of primary CCS of the pubic bone resembling SRCT. This ambiguous appearance underscores the difficulties encountered during the histological diagnosis of this rare variant of CCS.

**Conclusion:**

Awareness of primary CCS of the bone is clinically important for accurate diagnosis and management when the tumor is located in unusual locations such as the pubic bone and when the translocation t(12; 22)(q13; q12) is absent.

## Background

Clear cell sarcoma (CCS) of soft tissue was formerly known as malignant melanoma of soft tissues because of the presence of melanin pigmentation and (pre-)melanosomes in a significant percentage of these tumors. CCS was originally described by Enzinger in 1965, and it has become a well-defined clinicopathological entity since then [1]. CCS and malignant melanoma share overlapping immunohistochemistry with regard to the melanocytic markers HMB45, S100, and Melan-A. However, CCS generally lacks melanoma-associated *BRAF* mutations [2-4]. In addition, the translocation t(12; 22)(q13; q12) is specific only to CCS [5] and results in fusion of *EWS* (22q12) and *ATF1* (12q13). Therefore, although these neoplasms are closely related, they are now considered to be distinct entities. However, the translocation is apparently detectable only in 50%–70% of CCS cases. Absence of the detectable *EWS/AFT1* rearrangement may occasionally lead to erroneous exclusion of a translocation-negative CCS. Therefore, histological assessment is essential for the correct diagnosis of CCS [6]. In addition, CCS accounts for <1% of soft tissue sarcomas, and this tumor is so rare that there are no standard regimens. It typically involves tendons and aponeuroses of young adults. Primary CCS of the bone is exceedingly rare. Only a few cases of primary CCS arising in the ulna, metatarsals, ribs, radius, sacrum, and humerus have been reported [7-9], and, to the best of our knowledge, CCS arising in the pubic bone has never been reported.

## Case presentation

**Figure 1:**
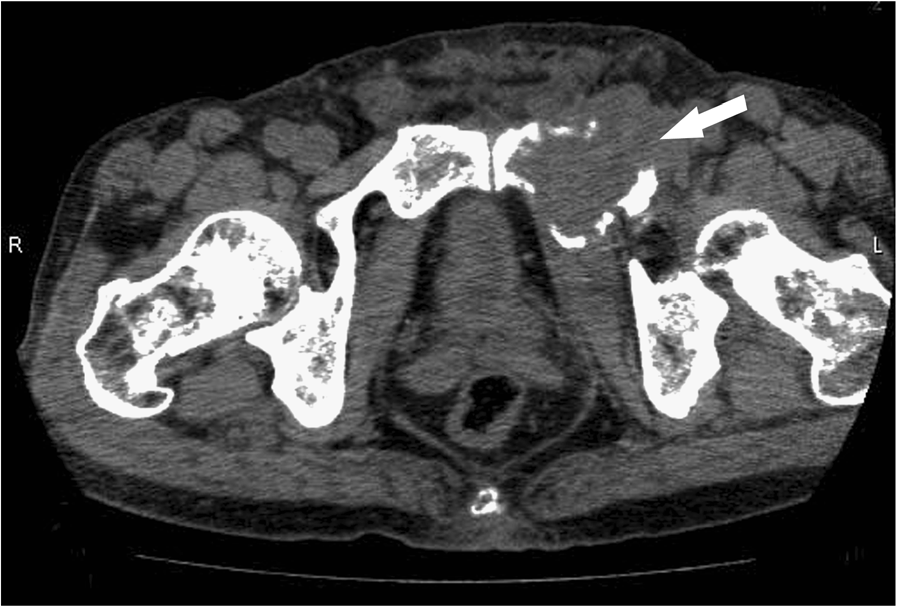
**Axial computed tomography of the pelvis demonstrating a solitary, lytic, expansile lesion in the left superior pubic ramus**.

**Figure 2:**
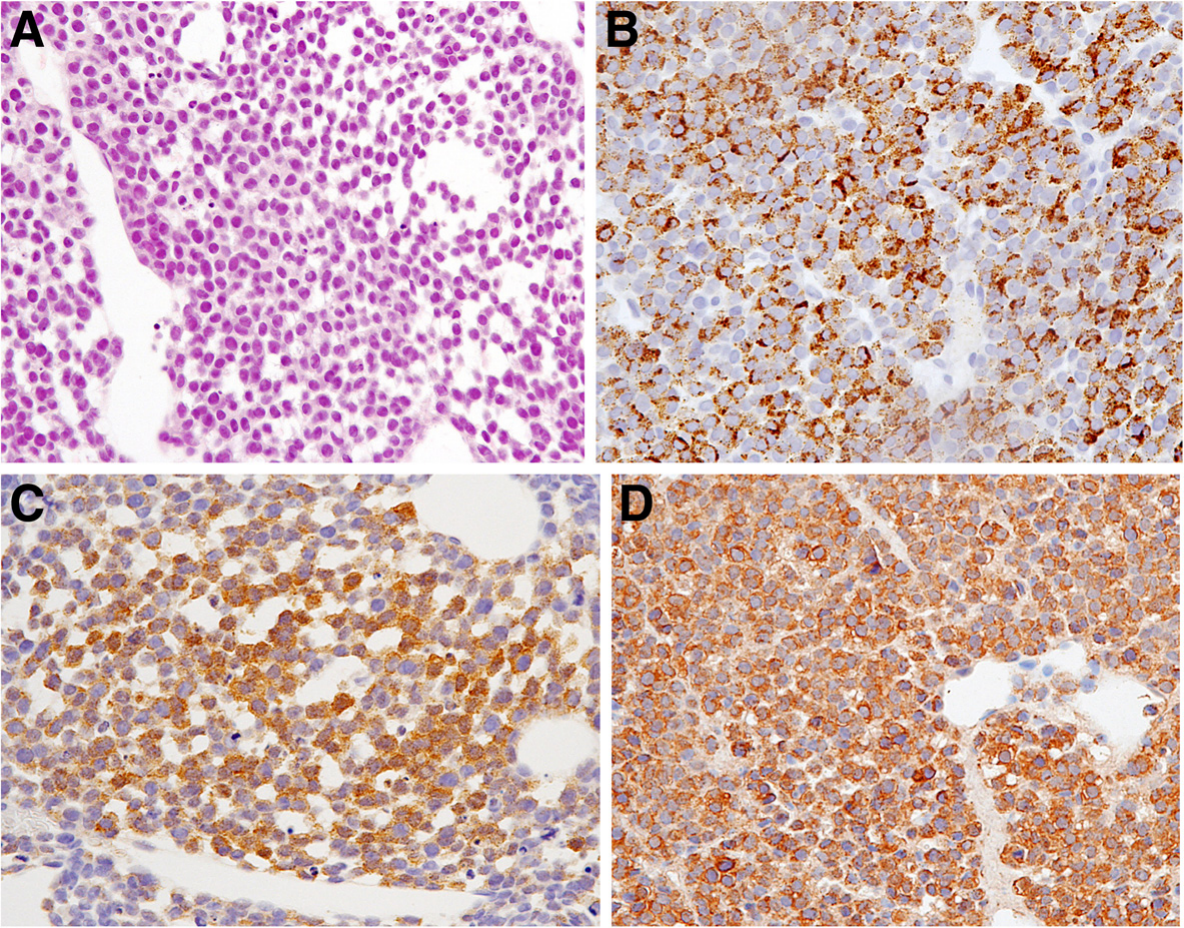
**(A) Diffuse infiltration of small round cells with inconspicuous nucleoli and scanty cytoplasm (hematoxylin and eosin stain; objective magnification, ×40).** (**B**) Abnormal cells positive for HMB45 (objective magnification, ×40). (**C**) Abnormal cells positive for S100. (objective magnification, ×40). (**D**) Abnormal cells positive for Melan-A. (objective magnification, ×40).

An 81-year-old man presented at our institution with right-sided groin pain since 2 months. He had no history of trauma, infection, or constitutional symptoms. Radiological examination on first admission revealed an expanding destructive lesion in the left superior pubic ramus. A technetium-99 m bone scan demonstrated activity in the left superior pubic ramus, and computed tomography (CT) revealed a mass encasing the left superior pubic ramus (Figure 1). Needle biopsy of the pubic bone was then performed. Histological examination of the biopsy specimen revealed monomorphic small cells arranged predominantly as a diffuse sheet. The cells had round hyperchromatic nuclei, inconspicuous nucleoli, and scant cytoplasm. These findings were indicative of small round cell tumor (SRCT; Figure 2A). On immunohistochemical staining, the tumor cells were positive for HMB45 (DAKO, Carpenteria, CA, USA; Figure 2B), S100 (DAKO, Carpenteria, CA; Figure 2C), and Melan-A (Novocastra, Newcastle-Upon-Tyne, UK; Figure 2D), whereas they were negative for cytokeratin (AE1/AE3) and epithelial membrane antigen. Fluorescence *in situ* hybridization analysis of the fusion signal of *EWS* located on chromosome 22q12 yielded negative results. *BRAF* (exons 11 and 15) mutation analysis by direct sequencing revealed the absence of mutations [4]. Although the biopsy specimen showed no obvious evidence of melanoma pigments, these findings suggested malignant melanoma or CCS. Whole-body CT, positron emission tomography (PET)/CT, and ^67^Ga-citrare scintigraphy were performed to exclude the possibility of metastases of malignant melanoma to the bone. The patient was referred to a dermatologist for evaluation of primary cutaneous malignant melanoma. All efforts to find a primary lesion elsewhere were unsuccessful. Eventually, we concluded that this was not a metastatic lesion of malignant melanoma but primary CCS of the pubic bone. Because of the patient’s advanced age, he and his family refused surgery as well as local radiation therapy; instead, they opted for chemotherapy. The patient was administered DAV chemotherapy (a regimen of dimethyl triazeno imidazole carboxamide, 1-[4-amino-2-methyl-5-pyrimidinyl]-methyl-3-[2-chloroethyl]-3-nitrosourea hydrochloride, the available substitute for bischloroethylnitrosourea, and vincristine) [10,11]; however, he died of progressive disease.

## Discussion

CCS typically presents a uniform, nested-to-fascicular growth pattern. Tumor cells are polygonal or spindle-shaped with abundant cytoplasm. Less common morphologic variations include spindle-cell arrangement, marked pleomorphism, solid-cell aspect, microcystic aspect, and presence of myxoid stroma [12].

SRCT comprises heterogeneous neoplasms comprising relatively small, round-to-oval, closely-packed, undifferentiated cells with a high nuclear/cytoplasmic ratio, scant cytoplasm, and round nuclei with evenly distributed, slightly coarse chromatin and small or inconspicuous nucleoli. SRCT comprises a group of highly aggressive malignant tumors [13]. Despite the similar morphology of CSS and SRCT under light microscopic examination, the latter differs from the former in that it includes pathological entities from vastly different lineages, including epithelial tumors such as small-cell carcinoma (poorly-differentiated neuroendocrine carcinoma); mesenchymal tumors such as malignant solid neoplasms of childhood and other small round-cell sarcomas, and tumors with overlapping features such as lymphoma and melanoma [14]. Small-cell malignant melanoma with the appearance of SRCT is one of the recognized rare variants of malignant melanoma, and it is frequently documented as a complication of congenital melanocytic nevi [15], as a childhood neoplasm [16], or as a tumor of mucosal origin [17]. However, CCS resembling SRCT has not been previously reported.

To the best of our knowledge, this is the first case report of primary CCS of the pubic bone resembling SRCT. This ambiguous appearance underscores the difficulties encountered during the histological diagnosis of this rare variant of CCS. Awareness of primary CCS of the bone is clinically important for accurate diagnosis and management when the tumor is located in unusual locations such as the pubic bone and when the translocation t(12; 22)(q13; q12) is absent.

### Consent

Written informed consent for publication of data was obtained from the patient.

## Abbreviations

CCS: Clear cell sarcoma; CT: Computed tomography; SRCT: Small round cell tumor; PET: Positron emission tomography.

## Competing interest

The authors declare that they have no competing interests. We have no personal or financial conflicts of interest related to the preparation and publication of this manuscript.

## Authors’ contributions

SN was involved in conception and design of the study, interpretation of data, and writing of the manuscript. All authors read and approved the final manuscript.

## Pre-publication history

The pre-publication history for this paper can be accessed here:

http://www.biomedcentral.com/1471-2407/12/538/prepub
